# miR-23b mediates TNF-α-Inhibited Osteogenic Differentiation of Human Periodontal Ligament Stem Cells by Targeting Runx2

**DOI:** 10.7150/ijms.64312

**Published:** 2021-09-09

**Authors:** Xuefei Sun, Mingwei Li, Jinghao Ban, Zhidan Li

**Affiliations:** 1 Key Laboratory of Shaanxi Province for Craniofacial Precision Medicine Research, College of Stomatology, Xi'an Jiaotong University, Xi'an, China.; 2 Clinical Research Center of Shaanxi Province for Dental and Maxillofacial Diseases, Department of Endodontics, College of Stomatology, Xi'an Jiaotong University, Xi'an, China.; 3 Department of Pediatric Dentistry, Nanjing Stomatological Hospital, Medical School of Nanjing University, 30 Zhongyang Road, Nanjing, 210008, China.; 4 State Key Laboratory of Military Stomatology & National Clinical Research Center for Oral Diseases & Shaanxi Clinical Research Center for Oral Diseases, Department of Preventive Dentistry, School of Stomatology, The Fourth Military Medical University.

**Keywords:** miR-23b, TNF-α, Runx2, hPDLSCs, Osteogenic differentiation

## Abstract

Periodontitis is the most prevalent oral infection disease, which causes the destruction of periodontal supporting tissues and eventual tooth loss. This study aimed to investigate the molecular mechanism of miRNA-23b (miR-23b) in regulating the osteogenic differentiation of human periodontal ligament stem cells (hPDLSCs) in an inflammatory environment. Results revealed that tumor necrosis factor-α (TNF‐α), a notoriously inflammatory cytokine, remarkably attenuated the osteogenic differentiation of hPDLSCs, which were partially rescued by SKL2001 (Wnt/β-catenin agonist). We further explored the underlying roles of miRNAs involved in TNF-α-inhibited osteogenesis of hPDLSCs. The miR-23b significantly increased with TNF-α stimulation, which was abolished by SKL2001. Similar to the effect of TNF-α, miR-23b agonist (agomir-23b) dramatically reduced the expression of runt-related transcription factor 2 (Runx2) and suppressed the osteogenic differentiation of hPDLSCs. The inhibition of miR-23b significantly increased Runx2, which is the major transcription factor during osteogenesis, thereby indicating that miR-23b was an endogenous regulator of Runx2 in hPDLSCs. Bioinformatic analysis and dual luciferase reporter assays confirmed that Runx2 was a target gene of miR-23b. Furthermore, the gain function assay of Runx2 revealed that the Runx2 overexpression efficiently reversed the suppression of the osteogenic differentiation of hPDLSCs with miR-23b agonist, suggesting that the suppressing effect of miR-23b on osteogenesis was mediated by Runx2 inhibition. Our study clarified that miR-23b mediated the TNF-α-inhibited osteogenic differentiation of hPDLSCs by targeting Runx2. Therefore, the expanded function of miR-23b in the osteogenesis of hPDLSCs under inflammatory conditions. This study might provide new insights and a novel therapeutic target for periodontitis.

## Introduction

Periodontitis is usually sustained from microorganisms, such as *Porphyromonas gingivalis*,* Treponema denticola*, and* Tannerella forsythia*, in the oral cavity localized in gum plaques. Studies on the pathogenesis of periodontitis have found that the influence of inflammatory microenvironments on patients with periodontitis inhibits the proliferation and osteogenesis of hPDLSCs and affects the remodeling and regeneration of periodontal supporting tissues 1. Among sophisticated pro-inflammatory cytokines, TNF-α is an important proinflammatory factor that can be rapidly secreted by T lymphocytes and macrophages 2. TNF-α suppresses other origins of the osteogenic differentiation of mesenchymal stem cells (MSCs) and bone regeneration 3, 4. TNF-α negatively mediates Wnt signaling to inhibit the osteogenic differentiation of bone marrow mesenchymal stem cells (BMSCs) 5, 6. TNF-α exerts a destructive effect on the osteogenic differentiation of periodontal ligament stem cells (PDLSCs) 7, 8. Therefore, TNF-α-targeted therapy may be essential for the osteogenic effect and periodontal regeneration of PDLSCs in an inflammatory microenvironment.

hPDLSC therapy has gradually become a novel periodontitis treatment because of their biocompatibility, immunomodulatory properties, multi-differentiation ability, and self-renewal properties 9. Previous studies demonstrated that hPDLSCs implanted with 3D scaffold materials can hasten the restoration of alveolar bone defects in animal models 10, 11. However, inflammatory microenvironments in periodontitis go against the desired periodontal tissue regeneration by hPDLSCs. Furthermore, hPDLSCs in the source of patients with periodontitis demonstrate an impaired osteogenic status, including the reduced expression of the osteoblast gene and a decrease in calcified nodule deposits and ALP activities 12. Therefore, improving the osteogenic differentiation ability of hPDLSCs against the inflammatory environment of periodontitis is the key to obtaining satisfactory therapeutic effects.

MicroRNAs (miRNAs) are post‑transcriptional regulators in gene expression that play a key role in regulating various biological and pathological processes. The effects of miRNAs on the osteogenic differentiation of other cell types have been studied by modulating miRNA functions, suggesting that miRNA regulation is important in this biological process 13. Reportedly, miR-23b regulates the balance between the differentiations of adipocytes and osteoblasts in BMSCs 14. In addition, the ectopic expression of miR-23b inhibits the osteogenetic effect by targeting Runx2 in BMSCs 15. However, the regulatory role of miR-23b in the osteogenic differentiation of hPDLSCs remains ambiguous, especially in an inflammatory environment. In the current study, we investigated the expression of miR‑23b and its function in the osteogenesis of TNF-α-inhibited hPDLSCs through the transfection of the exogenous agomir and antagomir of miR‑23b. In brief, miR-23b is involved in the TNF-α-inhibited osteogenesis of hPDLSCs via the mechanism of Runx2 inhibition. Our findings elucidated the regulatory role of miR-23b in the osteogenic differentiation of hPDLSCs in an inflammatory microenvironment and afforded a novel underlying therapeutic molecule target for periodontitis.

## Materials and methods

### Cell culture and transfection

PDLSCs were isolated and cultured as previously described 16. Informed consent was obtained for the acquisition of the teeth from male patients aged 18-22 years. All the experimental procedures were approved by the ethics committee of Xi'an Jiaotong University Stomatology Hospital. For miRNA transfection, the component was mixed in serum‑free α-MEM and transfected in complete α-MEM. The miRNA agomir (miR‑23b agomir), antagomir (miR‑23b antagomir), and negative controls of miR‑23b (miR‑23b NC; Ribo Bio Co., Ltd., Guangzhou, China) were transfected into PDLSCs by using Lipofectamine2000 (Invitrogen, USA) in accordance with the manufacturer's instructions. The miRNA sequences were as follows: miR‑23b agomir, 5′-AUCACAUUGCCAGGGAUUACC-3′; miR‑23b antagomir, GGUAAUCCCUGGCAAUGUGAU; and miR‑23b NC, 5′-UUGUACUACACAAAAGUACUG-3′.

### Flow cytometry analysis

The immunophenotype of PDLSCs was detected through flow cytometry in accordance with the manufacturer's instructions (BD Bioscience, USA). The following antibodies were used: MSC-positive markers (Stro1-PE, CD105-PE, and CD29-PE) and MSC-negative markers (CD34-PE and CD45-PE). Cells were digested with trypsin and suspended in a PBS solution before they were incubated with their respective antibodies at 37 °C for 30 min in the dark. They were then washed with PBS thrice and detected through flow cytometry.

### RNA purification and quantitative real-time PCR

Total RNA was extracted by using TRIzol reagent (Invitrogen, USA) in accordance with the manufacturer's instructions. First‑strand cDNA was synthesized from 1 μg of the total RNA by using a PrimeScript™ RT reagent kit (Takara, Japan). The amplification conditions were set as follows: denaturation at 95 °C for 10 s, 40 cycles denaturation at 95 °C for 10 s, annealing at 60 °C for 30 s, and a final extension step at 70 °C for 5 min. Quantitative real‑time PCR (qRT‑PCR) was analyzed by using the 2^-∆∆Ct^ method. GAPDH or U6 was used as the endogenous normalization control.

### Western blot analysis

The cells were washed with ice-cold PBS and lysed in a radio-immunoprecipitation assay buffer. Then, the protein sample was separated through 10% SDS-PAGE and transferred to PVDF membranes (Millipore, Germany). The membranes were blocked in 5% nonfat milk for 1 h and then incubated with primary antibodies (1:1,000; Abcam, UK) against Osterix, Runx2, p-GSK3β, GSK3β, β-catenin, and GAPDH (1:5,000; Abcam, UK) overnight at 4 °C. Afterward, they were incubated with secondary antibodies (Proteintech, USA) for 1 h, detected through chemiluminescence, and quantified by using ImageJ.

### Alizarin red staining and oil red O staining

Osteogenic differentiation was induced in cells in an osteogenic differentiation medium composed of α-MEM, 10 mM β-glycerophosphate, 0.1 μM dexamethasone, and 50 μg/mL ascorbic acid (Sigma, USA) for 3 weeks. The adipogenic differentiation medium composed of α-MEM, 0.5 mM IBMX, 200 μM indomethacin, 1 μM dexamethasone, and 10 μg/mL insulin (Sigma, USA). Then, the cells were fixed with 4% paraformaldehyde for 30 min and rinsed with PBS before 0.1% alizarin red staining (ARS; pH 4.2) or 0.1% oil red O staining at room temperature for 30 min. They were rinsed again with PBS while being gently agitated. It was dissolved with 10% cetylpyridinium chloride and analyzed by using a spectrophotometer at 562 nm absorbance to quantify ARS.

### Runx2 3′-UTR cloning and luciferase assay

The 3′-UTR fragment of Runx2 containing the wild-type or mutant miR-23b binding sites was synthesized, cloned into the downstream of the luciferase coding sequence in pmirGLO plasmids (Promega, USA), and designated as Runx2-WT or Runx2-MUT. The cells were co-transfected with miR-23b or a vector, together with *Renilla* plasmid (Promega, USA), and Runx2-WT or Runx2-MUT reporter transfected with Lipofectamine 2000 reagent (Invitrogen, USA). The luciferase activity was detected by using the luciferase reporter assay system (Promega, USA) after 48 h of transfection. The firefly luciferase activity was normalized to *Renilla*.

### Construction of Ad‑Runx2 and Ad‑EV

Adenoviruses encoding Runx2 (Ad-Runx2) and empty plasmid (Ad-EV) were constructed by GenePharma (Shanghai, China). The cells were transfected with Ad-Runx2 and Ad-EV at the indicated MOI in accordance with the manufacturer's instructions. After 72 h of lentivirus infection, stable transfectants were exposed to puromycin (Sigma-Aldrich, USA).

### Statistical Analysis

Statistically significant differences were determined using two-tailed Student's t-test or ANOVA. Data represented the mean ± SD of at least n=4 independent experiments. *p<0.05, **p<0.01, and ***p<0.001. ns, not significant.

## Results

### Identification of hPDLSCs

**Figure 1:**
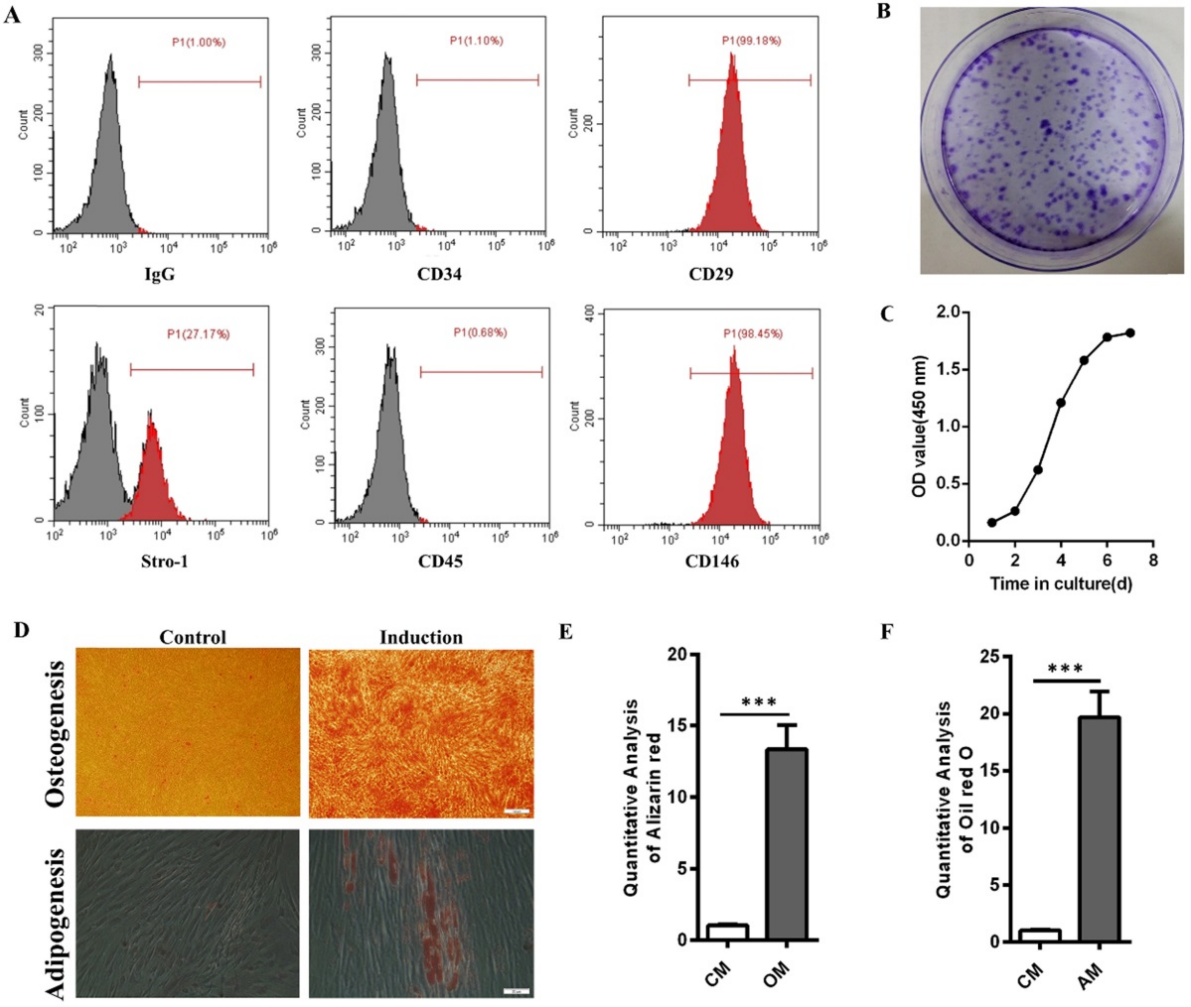
** Identification of hPDLSCs. (A)** Cell marker expression was measured through flow cytometry. **(B)** The colony-forming ability of hPDLSCs was detected by crystal violet staining. **(C)** The cell growth curve was obtained through spectrophotometry. **(D)** The osteogenesis and adipogenesis capabilities of hPDLSCs were determined through Alizarin red and oil red O staining, respectively. Scale bars, 100 µm (upper) and 20 µm (bottom). **(E, F)** Alizarin red staining and oil red O staining were quantitatively analyzed. CM, control medium; OM, osteogenic medium; AM, adipogenic medium.

Flow cytometry results showed that hPDLSCs highly expressed the mesenchymal stem cell markers CD29, CD146, and Stro-1 but scarcely expressed the leukocyte marker CD45 and the hematopoietic marker CD34 (Fig. 1A). Crystal violet staining and cell growth curve manifested the colony formation efficiency and self-renewal ability of hPDLSCs (Fig. 1B and C). The multidirectional differentiation ability of hPDLSCs was identified through Alizarin red and oil red O experiments. Most cells formed mineralized calcium deposits after they were cultured in OM for 3 weeks. Oil red O staining results indicated that lipid droplets were formed in hPDLSCs after 3 weeks of adipogenic induction. By contrast, few mineralized calcium deposits and lipid droplets were observed in the control groups (Fig. 1D-F). These data verified that the isolated hPDLSCs possessed the abilities of self-renewal and multilineage differentiation.

### TNF-α inhibits the osteogenic differentiation of hPDLSCs and induces the miR-23b expression via the Wnt/β-catenin pathway

**Figure 2:**
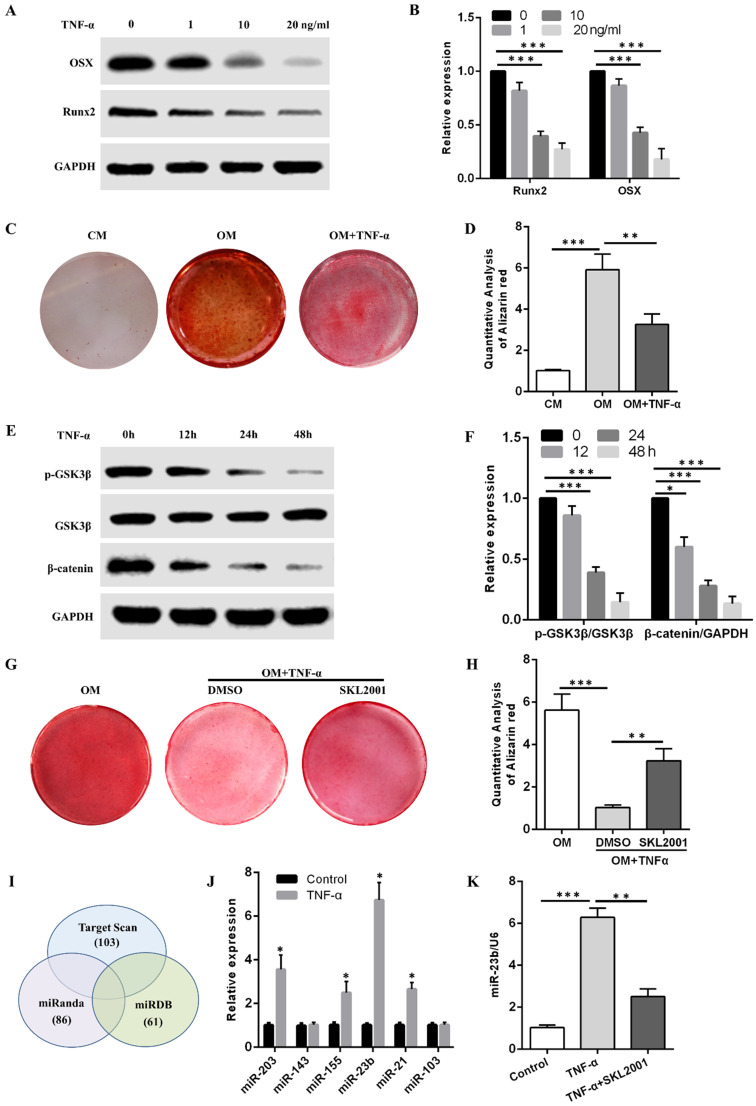
** TNF-α inhibits osteogenesis and upregulates miR-23b expression via the Wnt/β-catenin pathway. (A, B)** The expression of OSX and Runx2 protein in hPDLSCs with TNF-α for 1 week. **(C, D)** Alizarin red staining of the osteogenic differentiation ability of hPDLSCs under stimulation with or without TNF-α at 10 ng/mL. **(E, F)** Western blot analysis of p-GSK3β, GSK3β, and β-catenin expression levels in hPDLSCs treated with TNF-α in the osteogenic medium at different time points. **(G, H)** Alizarin red staining of the osteogenic differentiation ability of hPDLSCs under TNF-α (10 ng/mL) stimulation with or without SKL2001 (10 μM). **(I)** Three miRNA target prediction programs were used to predict Runx2-targeting miRNAs through bioinformatics. **(J)** The expression of the predicted miRNA in hPDLSCs stimulated with TNF-α for 24 h. **(K)** qRT-PCR of the miR-23b expression in hPDLSCs treated with TNF-α or in combination with SKL2001. CM, control medium; OM, osteogenic medium.

The Western blot results showed that TNF-α at concentrations of 1, 10, and 20 ng/mL reduced OSX and Runx2 protein levels in a concentration-dependent manner (Fig. 2A and B). Alizarin red S staining demonstrated that the calcium deposits obtained after osteogenic induction with 10 ng/mL TNF-α were fewer than those in the OM group (Fig. 2C and D). Alizarin red S staining results revealed that more calcium deposits were obtained in the OM + TNF-α + SKL2001 group than in the OM + TNF-α + DMSO group (Fig. 2G and H). They suggested that the activation of the Wnt/β-catenin signaling pathway dramatically rescued the inhibitory effect of TNF-α on the osteogenic differentiation of hPDLSCs. Western blot results showed that TNF-α in the osteogenic medium at different time points inhibited the p-GSK3β and β-catenin expression (Fig. 2E and F). Therefore, TNF-α inhibited the osteogenic differentiation of hPDLSCs through the Wnt/β-catenin signaling pathway.

Bioinformatic analysis was performed to predict miRNAs that might bind to the 3ʹUTR of *Runx2*. Specifically, *Runx2*-targeting miRNAs were filtered using three miRNA target prediction databases (i.e., miRanda, TargetScan, and miRDB). Six miRNAs, namely, miR-203, miR-143, miR-155, miR-23b, miR-21, and miR-103, targeted *Runx2* 3′-UTR through potential binding sites and were detected through qRT-PCR after TNF-α stimulation for 24 h. Considering the higher expression level of miR-23b among the above miRNAs, we chose miR-23b for the following experiments (Fig. 2J). The miR-23b was remarkably increased by TNF-α, which was abolished by SKL2001 (Fig. 2K). The results revealed that TNF-α inhibited the osteogenesis of hPDLSCs and induced the miR-23b expression via the Wnt/β-catenin signaling pathway in hPDLSCs.

### miR-23b suppresses the osteogenic differentiation of hPDLSCs by inhibiting the Wnt/β-catenin signaling pathway

**Figure 3:**
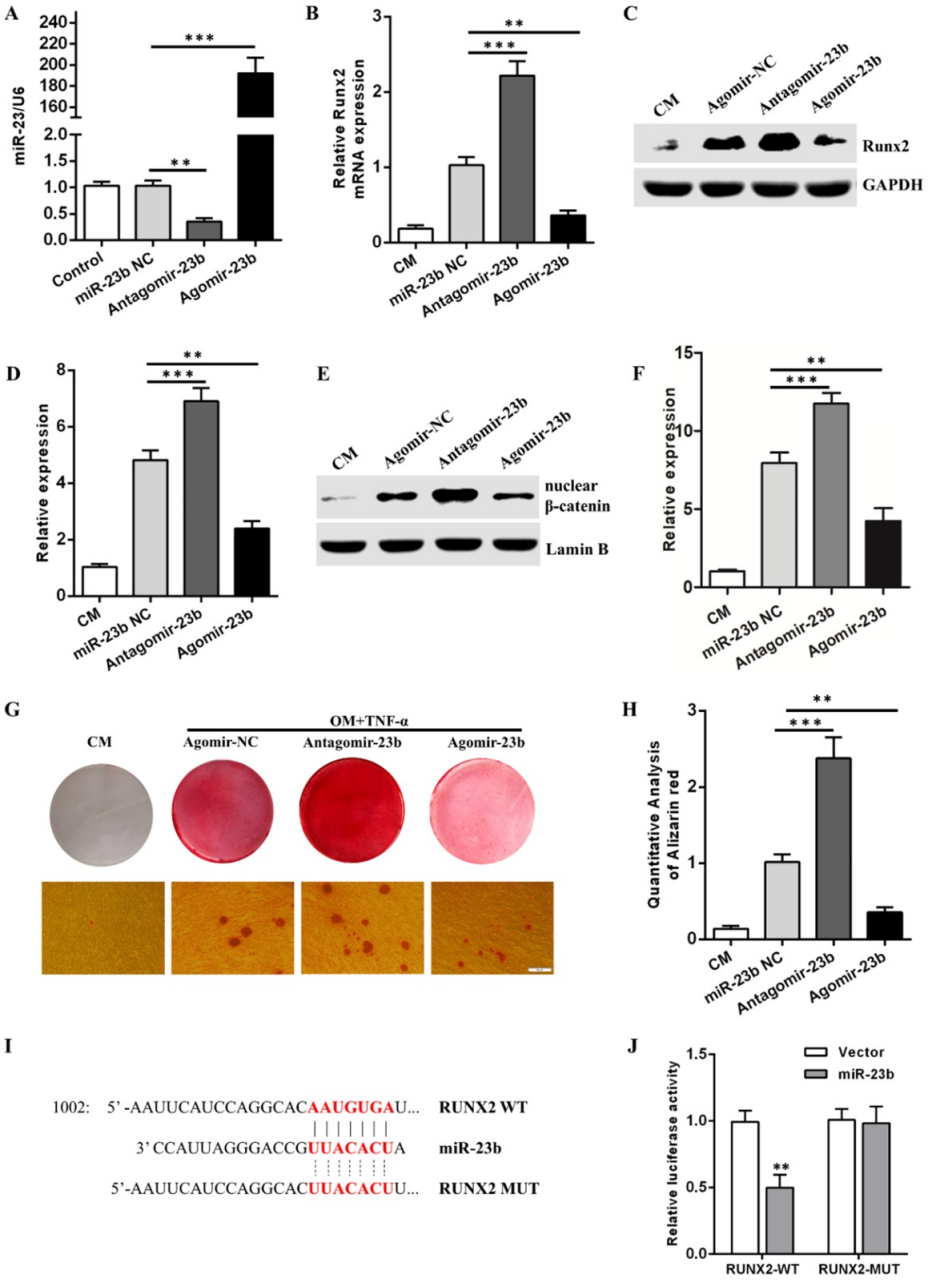
** miR-23b inhibits osteogenic differentiation and targets Runx2 in hPDLSCs. (A)** The expression of miR-23b was analyzed through qRT-PCR after the transfection of miR-23b. **(B)** qRT-PCR was used to detect the expression of Runx2 in hPDLSCs transfected with miR-23b agomir, antagomir, or agomir-NC. **(C, D)** Western blot analysis of the protein level of Runx2 in hPDLSCs that were transfected as described above. **(E, F)** Western blot analysis of the β-catenin protein level in hPDLSCs that were transfected as described above. **(G, H)** Alizarin red staining of hPDLSCs that were treated as described above. Scale bars, 50 μm. **(I)** Schematic of miR-23b indicating the binding site of the 3′-UTR of Runx2 (WT, wild type; MT, mutated). **(J)** The expression of the luciferase reporter activity. CM control medium, OM osteogenic medium.

Given that TNF-α suppressed the osteogenic differentiation of hPDLSCs, an approach combining a prompt augmentation of miR-23b, alizarin red S staining, qRT-PCR, and Western blot assay was performed to investigate the underlying role of miR-23b during the osteogenic differentiation of hPDLSCs. First, mir-23b was transfected in periodontal ligament cells and identified via qRT-RCR (Fig. 3a). Similar to the stimulation with TNF-α, the mRNA and protein expression levels of Runx2 were lower (Figs. 3B-D), and few calcium deposits were procured during the osteogenic differentiation of hPDLSCs after the miR-23b overexpression compared with the negative controls (Fig. 3G and H). The miR-23b overexpression significantly inhibited the β-catenin expression and vice versa (Fig. 3E and F). By contrast, the transfection of antagomiR-23b increased the Runx2 and β-catenin expression and the amount of calcium deposits, which further confirmed the above result (Fig. 3B-D, G and H). Collectively, miR-23b had a negative regulatory effect on the osteogenic differentiation of hPDLSCs via the inhibition of the Wnt/β-catenin signaling pathway.

### miR-23b directly targets Runx2 through a seed site in the 3'-UTR

Runx2 is an extremely crucial potential target gene of miR-23b. Sequence alignment displayed that the 3′-UTR of Runx2 matched with the miR-23b seed region (Fig. 3I). A dual luciferase report assay was used to verify the target genes and acquire further evidence that Runx2 was a direct target of miR-23b. Luciferase reporter constructs carrying the 3′-UTR of wild-type Runx2 (Runx2-WT) or mutant Runx2 (Runx2-Mut) were co-transfected with agomir-miR-23b (miR-23b) or agomir-NC (Vector) into hPDLSCs, respectively. miR-23b markedly reduced the luciferase activity of Runx2-WT but not Runx2-Mut (Fig. 3J). These results indicated that Runx2 was a direct target of miR-23b in hPDLSCs.

### Runx2 overexpression counteracts the miR-23b-mediated inhibition of osteogenesis

**Figure 4:**
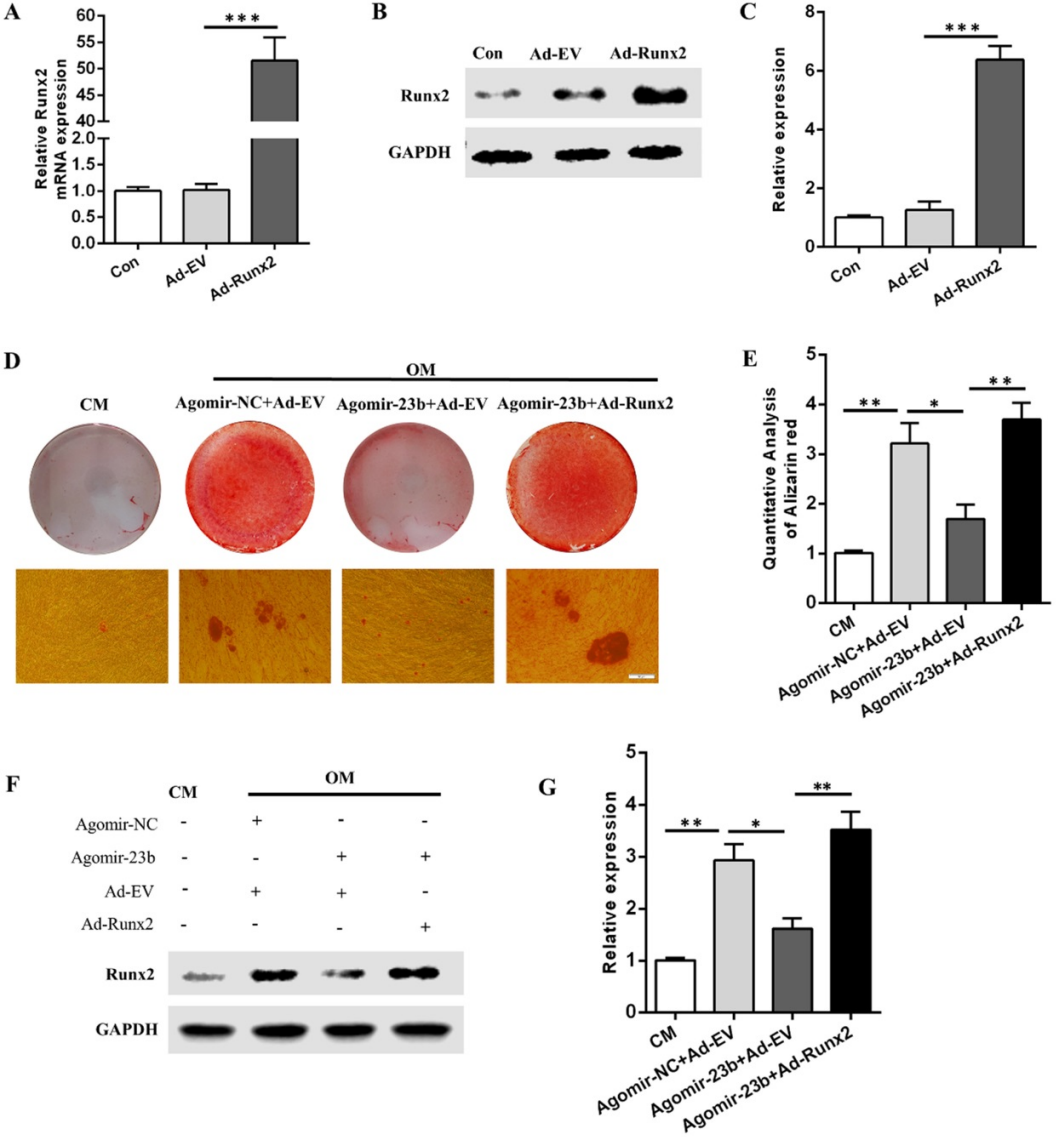
** Runx2 overexpression rescued the miR‑23b‑mediated inhibition of osteogenesis. (A)** The transfection effect of Runx2 adenovirus was detected through qRT-PCR. **(B, C)** The transfection effect of Runx2 adenovirus was detected through Western blot. **(D, E)** Alizarin red staining of hPDLSCs cultured in CM and OM. Scale bars, 50 µm. **(F, G)** Western blot of the Runx2 protein expression in hPDLSCs that were treated as described above.

To further explore the molecular mechanisms underlying the regulation of the osteogenic differentiation of hPDLSCs by Runx2 and mir-23b, we generated Runx2 overexpression adenovirus (Ad-Runx2). The mRNA and protein expression levels of Runx2 were detected via qRT-PCR and Western blot after infection with Ad-runx2 or adenovirus empty vehicle (Ad-EV). Undoubtedly, the Runx2 expression was comparatively upregulated at the transcriptional and post-transcriptional levels (Fig. 4A-C). The miR-23b-mediated inhibition of the osteogenesis of hPDLSCs was extremely reversed by the Runx2 overexpression (Fig. 4D and E). Western blot results demonstrated that the Runx2 protein expression level increased in Ad-Runx2 (Fig. 4F and G). These outcomes indicated that miR-23b mechanically suppressed the osteogenesis of hPDLSCs by inhibiting *Runx2*.

## Discussion

PDLSCs are promising seed cells for tissue engineering, especially in complete periodontal tissue regeneration 17, 18. The proliferative ability of PDLSCs is stronger than those of other odontogenic stem cells in bone regeneration, and PDLSCs have potential for periodontal tissue regeneration 19, 20. The inflammatory microenvironment in periodontitis is harmful to the regeneration and remodeling of periodontal tissues. Many other factors affect the growth and development of dental stem cells 21, 22. Mechanical stimuli induce proliferation and osteogenic differentiation and activate the proinflammatory cytokines of dental stem cells 22. Abnormal tooth anatomy influences the engineering application of periodontal ligament cells and tissues, whereas the improvement of a local tissue structure benefits local tissue regeneration 23, 24. Inflammation is the main factor that leads to the loss of periodontal tissues. During periodontitis development, the loss of bone tissues is closely related to the increase of TNF expression 25. The excessive release of TNF can cause periodontal and bone tissue damages, such as alveolar bone defect, alveolar fossa defect, and tooth support tissue defect 2. TNF‑α is a critical endogenous pro-inflammatory cytokine produced by monocytes and macrophages and negative regulator of osteogenic differentiation in response to bone-related inflammatory diseases, such as periodontitis and rheumatoid arthritis 26. Lacey et al. 27 showed that TNF‑α inhibits the expression of *ALP* and *Runx2* and the activity of ALP during the osteoblastic differentiation of BMSCs. Previous studies also demonstrated that TNF significantly inhibited the osteogenic differentiation of hPDLSCs through the NF-κB, MAPK, and mTOR signaling pathways 28, 29. In the present study, TNF-α at 1, 10, and 20 ng/mL reduced the OSX and Runx2 protein levels in a concentration-dependent manner. Moreover, the calcium deposits decreased after stimulation with TNF-α at 10 ng/mL under osteogenic induction. TNF-α at 10 ng/mL inhibited the p-GSK3β and β-catenin expression in the osteogenic medium at different time points. SKL2001 partially rescued the TNF-α-inhibited osteogenic differentiation of hPDLSCs. Thus, TNF-α inhibited the osteogenic differentiation of hPDLSCs through the Wnt/β-catenin signaling pathway. Consistent with our results, previous findings demonstrated that this pathway is widely involved in TNF-regulated osteogenic differentiation in various cells 30, 31. The results implied that miR-23b was involved in the negative regulation of TNF-α in the osteogenesis of hPDLSCs.

miRNAs are regulated by TNF-α in various cell types. Yang et al. 7 showed that TNF-α inhibits the osteogenic differentiation of hPDLSCs by suppressing the miR-21/Spry1 functional axis. miR-21 suppresses the osteogenic differentiation of PDLSCs by targeting Smad5 32. Wang et al. 33 showed that miR-155-3p mediates the TNF-α-inhibited osteogenic differentiation of cementoblasts by targeting Kctd1 and partially through the Wnt/β-catenin signaling pathway. Xia et al. 34 found that miR-203 inhibits the osteogenic differentiation of MSCs by regulating DKK1. As a new tumorigenic miRNA, miR-23b regulates osteogenic differentiation in various inflammatory microenvironments 15, 35. In the current study, miR-23b was dramatically upregulated in hPDLSCs in a TNF-induced inflammation microenvironment. The inhibition of miR‑23b promoted the protein and mRNA expression levels of Runx2 in TNF-α-inhibited osteogenic differentiation of hPDLSCs and enhanced the amount of the obtained calcium deposits and vice versa. The dual‑luciferase reporter assays clarified that miR-23b mediated the TNF-α-inhibited osteogenic differentiation of hPDLSCs by targeting Runx2. Intriguingly, Guo et al. 14 found that miR-23b regulated the balance between the differentiations of osteoblasts and adipocytes in BMSCs by inhibiting Tmem64. Tmem64 positively regulated osteoclast differentiation via the RANKL-mediated Ca^2+^ signaling pathway. Furthermore, Tmem64 regulated the differentiation of MSCs to adipogenesis rather than osteogenic differentiation by inhibiting β-catenin. Therefore, the negative regulation of miR-23b in MSC osteogenic differentiation might be due to the transfer of MSCs to adipogenesis, but it did not directly affect the osteogenic differentiation of MSCs. We confirmed that miR-23b negatively regulated the osteogenic differentiation of hPDLSCs directly by suppressing Runx2, thereby implying the crucial role of miR-23b in the regulation of bone metabolism.

We utilized three miRNA target prediction databases to perform a bioinformatic analysis to screen *Runx2*-targeting miRNAs. We predicted that miR-23b would conservatively bind to *Runx2* 3ʹUTR. Furthermore, we verified the direct binding between miR-23b and *Runx2* through dual‑luciferase reporter assays. The combination of miR-23b and *Runx2* 3′-UTR immensely attenuated the translation of *Runx2*, ultimately leading to osteogenesis suppression. Ad‑Runx2 was used for the confirmation of its considerable role in the process of miR‑23b-mediated TNF-α‑inhibited osteogenesis. As a critical osteogenic transcriptional factor, *Runx2* specifically binds to the regulatory regions of all major osteoblast-related genes to enhance the transcription of such genes 36. Notably, the overexpression of Runx2 in osteoblasts stimulates an increase in osteoclasts, thereby resulting in spontaneous fractures 37, 38. However, when the overexpression of Runx2 breaks the balance between osteogenic and osteoclastic differentiation, osteogenic differentiation likely occurs 39. Therefore, TNF-α induced the upregulation of miR-23b and inhibited the expression of Runx2. These processes might be involved in the novel mechanism underlying the TNF-α-inhibited osteogenic differentiation of hPDLSCs.

## Conclusion

**Figure 5:**
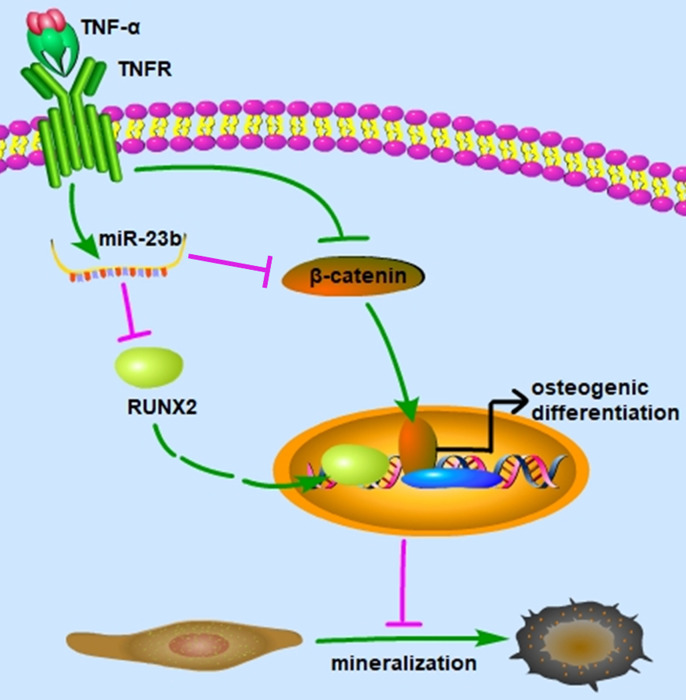
Schematic of this study. miR-23b targets Runx2 to mediate the TNF-α-inhibited osteogenic differentiation and mineralization; miR-23b, microRNA-23b; TNF-α, tumor necrosis factor α; TNFR, tumor necrosis factor receptor.

Our study elucidated that TNF-α suppressed the osteogenic differentiation of hPDLSCs at least partially via the canonical Wnt signaling pathway, and miR‑23b modulated the TNF-α-inhibited osteogenic differentiation of hPDLSCs by targeting Runx2 (Fig. 5). These findings indicated that miR‑23b could be a potential therapeutic target for periodontitis.
